# Artificial Intelligence and Citizen Science as a Tool for Global Mosquito Surveillance: Madagascar Case Study

**DOI:** 10.3390/insects16111098

**Published:** 2025-10-28

**Authors:** Ryan M. Carney, Farhat Azam, Karlene Gehrisch, Tanvir Bhuiyan, Lala S. Rafarasoa, Valéry Riantsoa, Russanne D. Low, Sarah Zohdy, Tovo M. Andrianjafy, Mamisoa A. Ramahazomanana, Ranto N. Rasolofo, Pradeep A. Subramani, Madison Ogbondah, Johnny A. Uelmen, Sriram Chellappan

**Affiliations:** 1 Department of Integrative Biology, University of South Florida (USF), Tampa, FL 33620, USA; karlenegehrisch@gmail.com (K.G.); uelmen@wisc.edu (J.A.U.J.); 2 Bellini College of AI, Cybersecurity and Computing, University of South Florida, Tampa, FL 33620, USA; farhatbinte@usf.edu (F.A.); tanvirrazin@gmail.com (T.B.); sriramc@usf.edu (S.C.); 3 Department of Medical Entomology, University of Antananarivo, Antananarivo 101, Madagascar; rafarasoalala@yahoo.fr (L.S.R.); riantsoav@gmail.com (V.R.); amthta@gmail.com (T.M.A.); ramahazomanana2@gmail.com (M.A.R.); rantorasolofo01@gmail.com (R.N.R.); 4 Institute for Global Environmental Strategies, Arlington, VA 22202, USA; rusty_low@strategies.org; 5 Entomology Branch, US Centers for Disease Control and Prevention, Atlanta, GA 30329, USA; sarahzohdy@gmail.com; 6 Global Health and Infectious Disease Research, University of South Florida, Tampa, FL 33612, USA; pannamalaisubramani@usf.edu (P.A.S.); madison541@usf.edu (M.O.); 7 Department of Population Health Sciences, University of Wisconsin (UW), Madison, WI 53575, USA

**Keywords:** *Anopheles*, artificial intelligence, citizen science, larva, machine learning, Madagascar, malaria, mobile applications, mosquito vectors, testes

## Abstract

*Anopheles stephensi* is an invasive and deadly malaria vector with the ability to use artificial containers as larval habitats. This ability is unique for malaria vectors in Africa and requires distinct surveillance strategies for early detection and rapid response. In this study, we trained a variety of artificial intelligence (AI) image recognition algorithms, using thousands of smartphone photos of laboratory-authenticated *An. stephensi* and seven endemic mosquito species, to develop a citizen science-friendly tool for *An. stephensi* detection. In Antananarivo, Madagascar, citizen science observations of >132 *Anopheles* spp. larvae from multiple artificial containers—including one closeup photo of a larva, from a tire—were submitted via NASA’s GLOBE Observer app in March 2020 and discovered years later. Given that genetic testing was no longer possible, this photo was used as a proof-of-concept to determine whether the AI species identification could be used on citizen science-generated images. The tire larva was classified as *An. stephensi* by all 11 species models, which yielded high accuracy and confidence (up to 99.34%) and included a false positive rate of <1%. Furthermore, explainable AI (XAI) heat maps led to the discovery of dark spots in abdominal segment VI corresponding to testes, corroborating a separate classification of the tire larva as male by the sex model. All available evidence suggests that AI image identification would have flagged this larva as a suspect *An. stephensi*, which could have been submitted to a molecular laboratory for further confirmation. Results demonstrate the power of integrating citizen science and AI—for which we provide free online tools—as a low-cost signal for malaria programs to confirm and respond to, and as complementary surveillance to fill the critical knowledge gaps in the distribution of invasive *An. stephensi* across Africa and beyond.

## Simple Summary

Malaria is a devastating mosquito-transmitted disease that infects over 250 million people and kills more than 600,000 every year. One mosquito of great global concern is *Anopheles stephensi*, an invasive species on the African continent. Unlike native malaria mosquitoes in Africa, this urban-adapted species can breed in artificial containers such as tires and buckets. Early detection of this mosquito is critical for rapid response to prevent increases in malaria; however, traditional surveillance and identification methods may overlook this invasive species. Therefore, we developed artificial intelligence (AI) programs to identify *An. stephensi* using photographed mosquito larvae (similar to the concept of facial recognition), since the most common method for *An. stephensi* surveillance is through the collection of larvae. As a practical proof-of-concept, these tools were used on a photo of a mosquito larva collected from a tire in Madagascar years earlier by local residents, who uploaded this and similar observations to a NASA app. While molecular confirmation is no longer possible on these long-discarded larval specimens, the successful use of AI programs to analyze mobile device photos provides a new opportunity to leverage citizen science for larval surveillance, even after the opportunity for physical collection has passed. Ultimately, this study demonstrates the value of integrating AI and citizen science to fight mosquito-borne diseases around the world.

## 1. Introduction

The presence and invasion of the *Anopheles* mosquitoes that transmit human malaria parasites are of accelerating global concern. The World Health Organization (WHO) reports that in 2020 alone, there were 241 million cases of malaria resulting in 627,000 deaths, including almost half a million children under the age of five [1]. In Madagascar specifically from 2019 to 2020, confirmed malaria cases and estimated deaths both unexpectedly doubled—to their highest numbers in at least two decades—with cases increasing by ~1 million and suspected cases reaching nearly 4 million [1]. From 2015 to 2020, Madagascar had by far the largest increase in both malaria incidence and mortality rates of any of the 11 east/south African countries with high transmission (approx. +38%, +43%; [1]). Malaria is a top-five cause of death in Madagascar, and the entire population of 33 million is at risk [2,3].

Malaria control in Africa faces serious challenges due to many factors, including the recent emergence of the invasive *Anopheles stephensi* (Liston, 1901) [4]. Although the full extent of *An. stephensi* distribution throughout the continent is unknown, confirmed specimens have so far been collected in Djibouti (2012), Ethiopia (2016), Sudan (2016), Somalia (2019), Nigeria (2020), Eritrea (2021), Kenya (2022), Ghana (2022) [4], and Niger (2024) [5]. In Djibouti from 2012 to 2020, reported malaria cases dramatically increased from only 27 to 73,535 [1], and epidemiological evidence has linked *An. stephensi* to malaria outbreaks in Djibouti and Ethiopia [6,7]. Exacerbating this epidemic crisis, *An. stephensi* is unusually susceptible to malaria parasites *Plasmodium falciparum* (Welch, 1897) and *P. vivax* (Grassi & Feletti, 1890) [8], and is highly resistant to pesticides [9]. The latter underscores the importance of vigilant surveillance and eliminating standing water habitats—in which mosquitoes oviposit (lay eggs) and develop as larvae and pupae—to prevent catastrophic malaria outbreaks. Indeed, it is estimated that *An. stephensi* will put an additional 126 million people in Africa at risk of malaria if left uncontrolled [10].

Thus, there is an urgent need for enhanced surveillance to identify the introductions and expanding distributions of *An. stephensi* across Africa [4], and control efforts will rely heavily on early detection of this invasive and fast-spreading species in new locations. Rapid response is especially critical before the mosquito population adapts and establishes itself beyond control. For example, five years after detection in Djibouti, *An. stephensi* was detected year-round, not just during the rainy season [11]. Six years after detection in Ethiopia, *An. stephensi* was directly associated with an urban malaria outbreak and comprised 97% of the adult mosquitoes collected [7]. This species has not yet been reported from Madagascar. However, among the coastal African countries, Madagascar is ranked as the sixth-highest risk of invasion by *An. stephensi* based on indices of maritime trade and habitat suitability [12], and the capital city of Antananarivo (pop. 2.5 M) is highly suitable for *An. stephensi* [10].

Unlike most endemic African malaria vectors that use natural breeding habitats such as puddles and rice paddies, *An. stephensi* is well-adapted to urban environments and can oviposit and develop in water within artificial containers. This ability is similar to that of *Aedes aegypti* (Linnaeus, 1762) and *Ae. albopictus* (Skuse, 1895), which are the principal vectors of dengue, yellow fever, and Zika. Indeed, one study in Ethiopia reported *Aedes* larvae in 40% of the sites where *An. stephensi* larvae were found [9]. Such artificial container-breeding mosquitoes are difficult to mitigate, as they opportunistically lay eggs in containers of all sizes, and locating these often-cryptic oviposition sites is labor-intensive for mosquito control professionals. Artificial containers also enable *An. stephensi* to persist throughout both dry and rainy seasons, a unique trait for malaria vectors which enables year-round transmission, confounds seasonal interventions, and is further enhanced by this species’ resilience to thermal extremes [13,14]. Due to challenges in collecting *An. stephensi* adults—specifically that human landing catches and CDC light traps are not successful in capturing *An. stephensi* [15] like they are in capturing routine *Anopheles* vectors—most efforts for the detection of this invasive species are based on larval surveillance [14,16].

For scaling such mosquito surveillance beyond traditional methods, citizen science (also known as community science) can be a useful and cost-effective approach [17,18,19]. Here, we describe a case study in Madagascar that integrates citizen science imagery with novel AI algorithms, as a complementary tool for larval surveillance.

## 2. Materials and Methods

### 2.1. GLOBE Observer

NASA/GLOBE Program’s GLOBE Observer (observer.globe.gov, accessed on 6 August 2025) is a mobile application with a Mosquito Habitat Mapper tool that enables citizen scientists to systematically describe and report observations of mosquito-breeding habitats (standing water) and associated larvae and pupae [20,21]. Available as a no-cost download to the public in 127 countries and 16 languages, the GLOBE Observer platform supports a suite of environmental monitoring tools including Clouds, Land Cover, and Trees, in addition to the Mosquito Habitat Mapper. Used together, these tools report data that allow scientists to understand the dynamics of mosquito populations across a varied landscape, ranging from urban to rural to natural environments.

Observations made using the GLOBE Observer tools are uploaded to the GLOBE database (1995-), which currently archives more than 270 million environmental measurements contributed by both GLOBE student participants and citizen scientists. Participation in the GLOBE Program is implemented through bilateral agreements between the U.S. government and governments of participating nations. All data archived by GLOBE are open and can be freely accessed online via application programming interfaces, through either the GLOBE Advanced Data Access Tool or the GLOBE Visualization System, an interactive map interface that allows users to filter, view, and download spatial data.

The Mosquito Habitat Mapper tool was beta-tested May–July 2017 and March–April 2018 in six communities in Brazil and Peru at high risk for Zika and dengue outbreaks, with support from the United States Agency for International Development program, Combating Zika and Future Threats: A Grand Challenge for Development. From 2018 to 2021, the follow-up GLOBE Zika Education and Prevention Project enlisted thousands of students, teachers, and communities to collect data on mosquitoes for a global mapping project and to connect with their community public health officials for disseminating educational information. More than 20 countries from Africa, Asia and the Pacific, and Latin America and the Caribbean were identified by the U.S. Department of State as high risk for Zika transmission and were supported in this effort through in-person training workshops and provisioning of supplies, including 60× clip-on lenses used to photograph mosquito larvae encountered during surveillance.

The Mosquito Habitat Mapper tool also includes a larva identification guide. As illustrated therein, *Anopheles* are relatively easy to identify at the genus level as they are the only mosquito larvae without a respiratory siphon.

### 2.2. Madagascar Observations

Given the recently escalating *An. stephensi* crisis, R.D.L. searched the Mosquito Habitat Mapper dataset by filtering observations using four criteria: within Africa, includes at least one photo of a larva, identified by the user as *Anopheles*, and collected from an artificial container; the resulting photos were downloaded and manually examined (see also [21]). One such photo of a potential *An. stephensi* was discovered, from years earlier in Madagascar.

**Figure 1:**
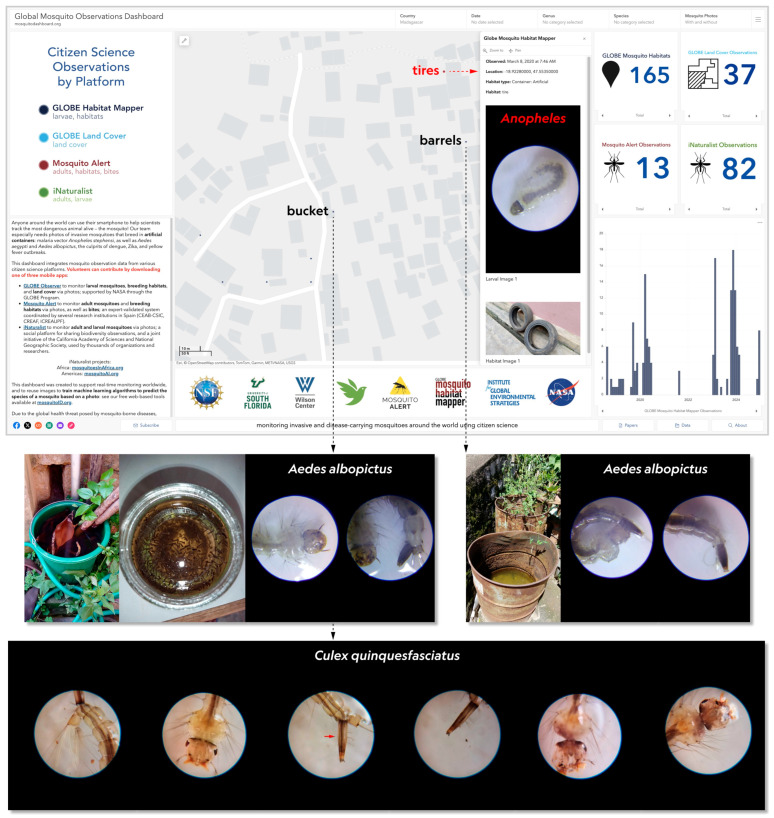
Global Mosquito Observations Dashboard (GMOD) (mosquitodashboard.org, accessed on 2 May 2025). Map displays Mosquito Habitat Mapper observations (dots), with labels denoting the three locations where >132 *Anopheles* spp. larvae were found in various artificial containers on 8 March 2020. Panel at right displays the tire containing 5 *Anopheles* spp. larvae, including the only closeup photo of an anopheline larva (“tire larva” herein; red dot on map). Note absence of siphon. Below are the original bucket and glass jar containing 486 transferred mosquito larvae—including >100 *Anopheles* spp. (not individually photographed) in addition to *Ae. albopictus* (note siphon) and *Culex quinquefasciatus* (note long siphon; arrow in third panel)—and the metal barrel containing 27 *Anopheles* spp. (not photographed) and 5 *Aedes albopictus*. Four of the remaining dots on the map represent ovitraps reported on 2 April 2020. At top right are counts of citizen science observations from each app platform, filtered to Madagascar; full details on the GMOD are available at [18,23].

This and nearby observations (Figure 1) were the result of larval monitoring and mitigation efforts targeting the artificial container-breeding Zika vector *Ae. albopictus* in urban areas, specifically the University of Antananarivo (UA) campus and its immediate surroundings, undertaken by the EntoAnkatso team led by L.S.R. during the GLOBE Zika Education and Prevention Project from October 2019–April 2020 [21,22]. As part of that project, during the 23rd GLOBE Annual Meeting in Detroit, Michigan, USA in July 2019, L.S.R. had been provided with approximately twenty 60× clip-on lenses that her team later used to photograph larvae in Madagascar.

On 8 March 2020, the aforementioned *Anopheles* spp. larva was collected from a tire and photographed (Figure 1) by L.S.R. and V.R. using a Samsung (Seoul, Republic of Korea) Galaxy Tab A tablet with a 60× clip-on lens. Per the app guidelines, this larva was late-stage, either third (L3) or fourth (L4) instar. One photo of two tires was also submitted. A total of five *Anopheles* spp. larvae were reported; no larvae from other genera were reported. Also observed were mosquito adults, but no eggs or pupae.

Within 100 m of the tires on that same day, L.S.R. and V.R. observed other *Anopheles* larvae in artificial containers. One observation included two metal barrels, one of which contained 27 *Anopheles* spp. and 5 *Ae. albopictus* (the latter reared to adulthood); two photos of an *Ae. albopictus* larva were uploaded (Figure 1). The other observation was a 10 L bucket containing 486 mosquito larvae, of which >100 were *Anopheles* spp.; two photos of an *Ae. albopictus* larva were uploaded (Figure 1). All of these larvae were transferred into a glass jar, a photo of which was included with six photos of a *Culex quinquefasciatus* (Say 1823) larva and uploaded from a different location on 26 March 2020. Both the barrel and bucket observations reported the presence of pupae and adults, but no eggs. The breeding habitats from the tire, barrel, and bucket observations were marked as eliminated, meaning that the water and/or artificial containers were removed.

Unfortunately, given that these observations were all from 2020, and the specimens were not preserved, morphological and molecular species confirmation was not possible. Another challenge was that only one *Anopheles* larva had been photographed close up, given the common practice that only one larva is examined through the app, and that the target for this surveillance was the Zika vector, *Ae. albopictus*. While larvae of the genus *Anopheles* are easy to identify (given the aforementioned diagnostic absence of a siphon), it is difficult to distinguish among the species. Additionally, there is no sufficient taxonomic key for identifying larval *An. stephensi* by the naked eye or at low magnification. As a solution, we leveraged a variety of AI techniques to provide empirical and explainable predictions for identifying the species and sex of the photographed *Anopheles* larva. For other recent work on larval classification using AI, see [20,24].

### 2.3. Larvae

#### 2.3.1. Centers for Disease Control and Prevention

As the *Anopheles* source for the Biodefense and Emerging Infections Research Resources Repository, the Malaria Research and Reference Reagent Resource (MR4) center provided mosquitoes reared according to the MR4 manual [25]. All species were authenticated for purity every five generations to ensure no contamination. The species and strains photographed included *Ae. aegypti* (KHW), *Ae. albopictus* (ATMNJ95), *An. arabiensis* (Patton, 1905) (Rufisque), *An. funestus* (Giles, 1900) (Fang for L3, Fumoz for L4), *An. gambiae* (Giles, 1902) (Ndokayo for L3, Kisumu for L4), *An. stephensi* (SDA-500), *Cx. quinquefasciatus* (JHB), and *Cx. tarsalis* (Coquillett, 1896) (Yolo). As described in Imaging below, every species was represented by 35 specimens each of L3 and L4 larvae, yielding at minimum 420 photos for each class (i.e., each combination of species × instar). Additionally, all photos from all specimens of *An. arabiensis* (Rufisque) and *An. stephensi* (SDA-500), along with new photos of STE2 and UCI strains of *An. stephensi*, were examined for the presence of dark spots in abdominal segment VI (35 specimens of each of the two instars, from each of the four strains).

#### 2.3.2. University of South Florida

Additional *An. stephensi* (Nijmegen) and *An. gambiae* (G3) were reared in walk-in chambers maintained at a constant temperature of 26 °C and a relative humidity of 80%, with 12 h light and dark photoperiod. The larvae were reared on an aquatic fish hatchery diet at a density of around 250 larvae per tray, holding 1.5 L of natural spring water. Upon maturation, the pupae were segregated for adult emergence in a secured mosquito cage, and on adult emergence, the mosquitoes were supplemented with 10% glucose solution through cotton pads. The adult mosquito colonies were maintained as F1 generations. For egg production to maintain the colony, 4- to 6-day-old female mosquitoes were fed with sheep blood by artificial membrane and provided with an ovitrap, a source of water for mosquito egg laying, two days post-blood meal [25]. Specimens of L4 *An. stephensi* (eight male, eight female) were also reared to adulthood and examined to confirm their sex, based on the presence/absence of dark spots in segment VI as observed in the larval stage.

#### 2.3.3. University of Antananarivo

In addition to the validation and testing datasets described below, we also evaluated species model specificity using wild L4 larvae collected in Antananarivo as part of our surveillance efforts [26]. One full body photo was taken and analyzed per specimen to emulate that of the tire larva, totaling 118 specimens/photos. These larvae were then identified morphologically after individually rearing to adulthood (e.g., *An. gambiae* s.l.) and/or by sequencing (i.e., *An. arabiensis*). The species and number of specimens/photos were *Ae. albopictus* (28), *An. arabiensis* (6), *An. gambiae* s.l. (48), and *Cx. quinquefasciatus* (36).

### 2.4. Imaging

**Figure 2:**
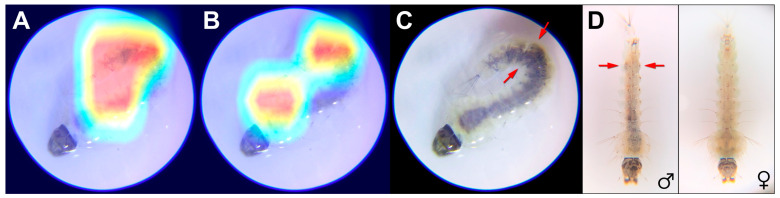
Tire larva photo with explainable artificial intelligence visualizations. Heat maps denote relative importance of individual pixels to the species classification, with warmer colors corresponding to higher weights. (**A**) Grad-CAM from the 6-class EfficientNet-B0 model (species confidence 99.34%), with a threshold of 185. (**B**) CAM from the 4-class EfficientNet-B0 model (species confidence 88.84%), with a threshold of 165. Note that these heat maps highlight segment VI where the testes are located. (**C**) Arrows denote testes. (**D**) Male and female L4 larvae of *An. stephensi* (Nijmegen strain), illustrating the presence (arrows) and absence of testes. Both specimens were photographed using a smartphone with a 60× clip-on lens, then reared to adulthood to confirm sex. Sharpening and/or improved contrast was applied to the images in (**C**,**D**).

All insectary and wild larvae were prepared and photographed (in dorsal view) in accordance with GLOBE Observer in-app instructions, using smartphones equipped with the same inexpensive 60× clip-on lens used in the 2020 observations and recommended by GLOBE (step-by-step guide is available in (Figure 2, File S1 [18]). Specifically, individual larvae were isolated from their water source using a pipette, dropper, or spoon and transferred to a white plate. Each larva was suspended in a small amount of water to ensure proper separation and visibility of the setae during photography. As needed to minimize its movement, the larva was chilled in its container using a surrounding ice bath beforehand, and/or repositioned using a toothpick. Insectary larvae were photographed using various Apple iPhones (CDC: 12, 12 Pro; USF: XR, 13 Pro Max) and wild larvae were photographed using a Blackview BV6200, iPhone 6, LG V50 and V50S ThinQ, Samsung Galaxy S7 Edge, Sony Xperia Z5 Compact, and Vivo Y21 (Apple: Cupertino, CA, USA; Blackview: Shenzhen, China; LG: Seoul, Republic of Korea; Sony: Tokyo, Japan; Vivo: Dongguan, China). As needed to improve focus, a washer was placed around the larva to slightly elevate the iPhone XR and 13 Pro Max. Per the aforementioned guide, the 60× clip-on lens itself was never extended on any phone, to standardize magnification.

For training, validating, and testing the species models, we used a total of 1680 photos from 560 unique specimens: 35 specimens from each of the two instars (L3, L4) of the eight species. At least two photos each were taken of three regions—full body, head/thorax, and terminal abdominal segments—and the most in-focus photo of each region was chosen. All models used these three photos per specimen, except for the genus (*Anopheles*, not *Anopheles*) and 16-class models, which used only the full body and head/thorax photos (1120 photos total for each; yielding 560 and 70 photos per class, respectively). The 3-class model combined L3 and L4 instars, yielding 210 photos per class. All other models thus used 105 photos per class (e.g., 1470 photos for the 14-class).

We split the dataset into training, validation, and testing images, maintaining the ratio 80:8:12. To enhance model reliability and robustness, we then increased the training dataset eight-fold (e.g., 9408 images for the 14-class) by using well-established test-time augmentation techniques: blur, brightness/contrast, crop, flip horizontal, flip vertical, rotate clockwise, rotate counterclockwise, and sharpen. For brightness/contrast augmentation, the code randomly choose one brightness value and one contrast value (from −0.2 to 0.1) and applied them to the image. We used the Python library Albumentations version 2.0.6 to apply a median blur to an image, effectively reducing noise while preserving edges. By setting the blur limit at 31 to 51 pixels, we achieved significant blurring and pronounced smoothing. Lastly, we manually cropped the original mosquito image with a rectangular box to remove all possible background while ensuring the entire mosquito body was contained.

For the sex model, we used a subset of the original *An. stephensi* photos, consisting of 264 photos from the CDC (for training and validation) and 98 photos from the USF (for testing) insectaries. These specimens were chosen based on the conspicuous presence (male) and absence (female) of dark spots (testes) in abdominal segment VI. We also took six photos each from the 16 additional L4 specimens (eight male, eight female) from the USF insectary that were reared to confirm their sex. From each reared male and female group, we used six specimens for training, one specimen for validation, and one specimen for testing. In total, this yielded 458 photos from 64 specimens (Table 1). For training, validation, and testing, we augmented our image data eight-fold. For each image, we first horizontally mirrored it. Then, from each of these two images, we generated three new images by rotating 90, 180, and 270°, yielding eight images in total. While building up the datasets, we worked with the original images. After an original image was placed in a certain dataset, the seven other versions of that image were kept in the same dataset.

### 2.5. Artificial Intelligence

We used these larval photos to train deep learning models, specifically convolutional neural networks (CNNs), which are effective for image classification. To identify the species of the tire larva, we trained EfficientNet [27] and Inception-ResNet-V2 [28] models (Table A1) with the hyperparameters shown in Table A2. For the former architecture, we trained B0 (5.3 M parameters) and B4 (19 M) networks, which are generally better suited to our training dataset sizes. Smaller networks like B0 are more suitable for datasets with fewer classes, whereas medium-sized networks like B4 tend to perform better by capturing more intricate features as class diversity increases. A model with a large number of parameters (e.g., B7 with 66 M) is optimal for large-scale datasets, but when trained on a small dataset tends to be overfitted and yields poor results on the testing dataset.

For visualization and validation, we utilized the explainable AI (XAI) techniques of class activation mapping (CAM; [29]) and Grad-CAM [30]. These approaches yield heat maps that illustrate the relative relevance of individual pixels to the classification. Heat maps were generated by combining the activation maps from the last convolutional layer with class-specific weights derived from either learned parameters (CAM) or gradient information (Grad-CAM). The resulting maps were then normalized, upsampled to the input resolution, thresholded, and superimposed on a grayscale version of the original image at 50% opacity.

To identify the sex of the tire larva, we used transfer learning, specifically an Xception [31] model pre-trained on the ImageNet dataset [32]. On top of the base model convolution layers, we added four dense layers (Table A3). The hyperparameters are shown in Table A4. 

Prediction confidence was calculated as the average classification probability (softmax output) across the 8× augmentation images of the tire larva. Initial evaluation of architectures also included ResNet50 for the species models and EfficientNet and VGG16 for the sex models, but these proved to be suboptimal and were not pursued further.

## 3. Results

Out of the 118 wild larvae we collected and reared in Antananarivo, the 16-class model misclassified as *An. stephensi* only one photo/specimen (of *Cx. quinquefasciatus*), yielding false positive rates of 0% for the *Anopheles* subset and <1% overall (0/54, 1/118). Notably, all 11 species models classified the tire larva as *An. stephensi* (Table 2). The highest level of confidence (99.34%) was achieved by the EfficientNet-B0 6-class model, with validation and testing accuracies of 96.30% and 95.83%.

The tire larva’s precise instar (L3 vs. L4) could not be ascertained by multiple mosquito entomologists. However, 6/8 (75%) of our species × instar models predicted L4 (L3 was predicted by the 4-class EfficientNet-B0 and 16-class models). Furthermore, there is a much higher confidence for the *An. stephensi* L4 vs. L3 classification by the highest-confidence 6-class model (Table 3) (82.56% vs. 16.78%) and especially the 8-class model (97.25% vs. 0.65%). There is also a much higher confidence for the *An. stephensi* classification by the 2-class L4 vs. 2-class L3 model (99.27% vs. 67.95%; Table 2).

The Grad-CAM/CAMs highlighted pixels of the tire larva—notably including abdominal segment VI—as most important to the species classification (Figure 2A,B, red). Additionally, the tire larva was classified as male with 100% confidence by our Xception sex model, with validation and testing accuracies of 95.44% and 84.89% (Table 4).

Informed by these results, we subsequently reexamined the original tire larva photo and discovered dark spots in segment VI where the heat maps were concentrated (Figure 2A–C). These spots are identical to the dark brown oval spots in segment VI that correspond to the testes of *An. stephensi* [33,34,35] (Figure 2D). Furthermore, we observed similar dark spots in segment VI in half of the L3 (53/105, 50.48%) and L4 (60/121, 49.59%) specimens examined from all four strains of *An. stephensi* (Nijmegen, SDA-500, STE2, UCI). Approximately one-quarter portion (32/113, 28.32%) of specimens with dark spots had a spot that appeared darker or only on one side. None of the 70 specimens of *An. arabiensis* (Rufisque) exhibited dark spots in segment VI. However, six specimens (9%) exhibited faint yellow localized there.

## 4. Discussion

### 4.1. Artificial Intelligence

The consensus of classifications, coupled with the high confidence, high accuracy, extremely low false positive rate, and XAI results, suggests that the identity of the tire larva is *An. stephensi* (although it is not possible to definitively confirm this without genetic analysis). Across multiple neural network architectures and models, EfficientNet yielded the best performance for classifying species compared to Inception-ResNet-V2 (aside from the 4-class models) and ResNet50, while Xception yielded the best performance for sexing larvae compared to EfficientNet and VGG16. Compared to Inception-ResNet-V2, EfficientNet trains faster and typically achieves higher performance on benchmark datasets commonly used to evaluate state-of-the-art models. The added complexity of EfficientNet is better suited for classifying species, as it has more layers to increase the receptive field and extract a richer diversity in image datasets. This architecture also employs a technique to uniformly scale all dimensions of depth, width, and resolution, enabling it to capture more fine-grained spatial patterns. By contrast, the Xception architecture employs depthwise separable convolutions, which is better tuned to learn a limited set of features at a specific location. In the context of sexing larvae, our Xception model is better equipped to learn the presence or absence of testes at segment VI (presumably yielding its superior classification performance), and the lower complexity is better suited for smaller datasets with only two classes (e.g., male and female).

The XAI results validated that the classifications were indeed based on the anatomy, and even revealed unexpected and useful anatomical information, demonstrating the utility of these techniques for mosquito larvae (as we have demonstrated for adults: Figure 6E [18]). The Grad-CAMs/CAMs that best highlighted the tire larva testes were from the all-*Anopheles* models (e.g., 6- and 4-class). We infer that this may be due to the other anatomical differences, such as the presence of a siphon, that distinguish the other genera included in the higher-class models. Adding more classes to the model also means that there are more parameters (necessitating more training optimization) and a bigger dataset with additional variation, which may make the multi-class classification task more complex and explain why better Grad-CAMs/CAMs were generated by the lower-class models. We also tried cropping the tire larva image, but the uncropped Grad-CAMs/CAMs were consistently better, presumably because uncropped images were used in 7/8ths of the training set.

Future work using XAI approaches and/or novel applications of other techniques such as geometric morphometrics [36] may prove similarly useful in revealing anatomical insights, and could potentially even inform future identification keys. Other promising practical applications include automated AI-enabled sex sorting in the larval stage, which would prove valuable for eco-friendly techniques for suppressing *An. stephensi* populations. While not yet established and available, these potential approaches include *Wolbachia* infection [37], genetic modification [38], and the sterile insect technique [39] (see also the heterospecific boosted version [40]). With respect to hardware, our future efforts will focus on further development and deployment of AI-enabled smart traps for targeting adult *An. stephensi* and other vector, invasive, and/or marked mosquitoes.

### 4.2. Sexing

Our XAI-enabled detection of testes in the tire larva provides independent corroboration of the male classification by the separate sex model. Such dark testes in certain species of mosquito larvae were first described and depicted in 1912, notably in *An. stephensi* and other Indian anophelines such as *An. culicifacies* (Giles, 1901) [35,41]. Proposed as a sexing marker, this feature was reported as most easily seen from the ventral side of abdominal segment VI, and consisting of a hard sheath that enclosed the testis and contained dark pigment. Rishikesh in 1959 (Figure 1 [34]) described and depicted this structure in laboratory colonies of Indian *An. stephensi* s.s. as a tough, outer envelope with a cytoplasm containing a “dense aggregate of yellow and dark pigment granules” (presumably xanthommatin and an ommin [42]).

More recently, [33] rediscovered these sexually diagnostic spots in both laboratory and field-collected specimens of *An. stephensi*, as well as *An. culicifacies* and *An. subpictus* (Grassi, 1899) (apparent in L3 and L4 but not in L1 and L2 of all species). This is interesting given that all three species have a type locality of India [43], from or around which they potentially originated, although each species belongs to a separate series of the subgenus *Cellia* [43]. 

With respect to the likelihood of the tire larva belonging to *An. culicifacies* or *An. subpictus*, neither have been reported in Madagascar [43]. Furthermore, *An. culicifacies* is typically found in non-urban settings such as streams and irrigation channels [44], and belongs to the Myzomyia series [43], thus if the tire larva were *An. culicifacies*, it presumably would have been classified by our models as the most closely related *An. funestus*. *Anopheles subpictus* belongs to the Pyretophorus series [43], thus if our tire larva were *An. subpictus*, it presumably would have been classified by our models as the more closely related *An. arabiensis* or *An. gambiae* s.s.

Across all four strains of *An. stephensi* herein, half of the 226 specimens exhibited dark spots corresponding to testes. Conversely, there was a complete absence of such dark spots among all 70 specimens examined of *An. arabiensis* (a species not documented in India). The faint yellow observed in segment VI among a small minority (9%) is interpreted as much less conspicuously pigmented testis sheaths in this species and/or strain. Additional research is required to ascertain the presence and degree of testis sheath pigmentation among *Anopheles* spp., as well as any influence of environmental or epigenetic factors.

### 4.3. Surveillance

Catalyzed by the initial AI classifications of the tire larva as a suspect *An. stephensi*, from July 2022–July 2023 we conducted follow-on systematic larval surveillance in all six districts of Antananarivo, starting at the locations of the 8 March 2020 observations; that separate study is detailed in [26]. To summarize: 2856 potential breeding habitats were inspected, 1886 of which were artificial containers (barrels, cut plastic canisters, flowerpots, metal containers, plastic bowls, plastic buckets, small plastic containers, tires, and used bottles). A total of 27,742 larval specimens were collected from three genera (*Aedes*, *Anopheles*, and *Culex*), with the most abundant species being *Cx. quinquefasciatus* (53%) and *Ae. albopictus* (26%). Of the 7290 larvae of the container-breeding species *Ae. albopictus*, 6446 (88%) were found in artificial containers, including 2186 from tires. Conversely, not a single one of the 1270 anopheline larvae was found in any of the 1886 artificial containers sampled, which included 626 tires, barrels, and buckets. No specimens of *An. stephensi* were detected. However, those systemic absences of any other anopheline larvae in artificial containers in 2022–2023, coupled with the presence in 2020 of >132 anopheline larvae in a tire (5), barrel (27), and bucket (>100), supports the hypothesis that the photographed tire larva could potentially represent the container-breeding species *An. stephensi.*

If any of these larvae detected in March 2020 do indeed represent *An. stephensi*, the absence of this species during the 2022–2023 surveillance could be due to the local population having died out (or having never become established in the first place). A contributing factor may have been the artificial container habitats being eliminated—not just those officially reported as eliminated herein and elsewhere [22], but the many others as part of the concentrated community efforts at that time to mitigate the container-breeding Zika vector, *Ae. albopictus*. Unlike *Aedes* and *Culex*, no other *Anopheles* larvae were observed in any artificial containers by the EntoAnkatso group after their 2019–2020 campaign.

Furthermore, the introduction of *An. stephensi* into the capital city of Antananarivo could have been due to international air travel or transport. Alternatively, it could be explained by transport of artificial containers or livestock from a seaport. It is worth noting that eggs of *An. stephensi* can survive in the absence of water for up to approximately two weeks [45]. Introduction could even have been caused by long-range easterly wind dispersal of adult mosquitoes from the nearest and main seaport Toamasina—as has been hypothesized for the isolated record of *An. stephensi* in Nigeria, which was >100 km from the nearest seaport and major airport and was not on a main road [46]. Three similarities shared with the *Anopheles* larvae in Madagascar herein are that these Nigerian specimens of *An. stephensi* (n = 14) were detected in only one area of the country (at two breeding sites, [47]), in the same year of 2020, and yet no further specimens of *An. stephensi* have been reported from thorough surveillance in Nigeria since [48].

Our team’s surveillance efforts in Madagascar also included expanding citizen science contributions through four local workshops at an elementary school and universities in Antananarivo and Antsirabe, translating educational and promotional materials into French, distributing hundreds of 60× clip-on lenses for photographing larvae using Mosquito Habitat Mapper, and distributing fliers promoting all the app platforms and GMOD. We are deploying similar strategies in other high-risk African countries as well, including the nearby island nation of Mauritius [45].

In total, >1500 lenses were provided to 18 African nations. We have also been encouraging and monitoring input across all three global citizen science platforms for mosquitoes: Mosquito Habitat Mapper, Mosquito Alert, and iNaturalist. For the iNaturalist platform, efforts included our project mosquitoesInAfrica.org (accessed on 15 August 2025)—designed to generate observations and identifications of *Anopheles* mosquitoes and particularly *An. stephensi* in Africa—which we launched as an annual campaign on 1 August 2022 [17]. During this campaign, we specifically monitored observations from Madagascar, which yielded a previously undescribed phenomenon of mosquito bite-induced color change in chameleon skin, underscoring the utility of citizen science in yielding serendipitous discoveries [49].

### 4.4. Recommendations

Global health organizations have long stressed that involvement of the community in the surveillance and mitigation of standing water breeding sites is critical to effective management of mosquito-borne diseases [50,51,52]. Indeed, a recent systematic review of community-based interventions in mosquito control demonstrated the positive impact of public engagement in reducing the prevalence of vectors and diseases in communities [53]. The case study reported herein demonstrates the importance of citizen science engagement not only for local health outcomes, but also for the research-creating tools that support global strategies to combat vector-borne diseases in the context of funding gaps and emergencies [54].

As mentioned above, larval surveillance is the main approach for detecting *An. stephensi*. The GLOBE Observer app’s Mosquito Habitat Mapper tool, put into the hands of volunteers in communities at risk of disease, can augment *An. stephensi* training, surveillance, and water source reduction; efforts that aid in local eradication and halting the expansion of this invasive and pesticide-resistant malaria vector [15]. Indeed, for citizen scientists, larval surveillance is easier than adult surveillance in many ways, as it does not require traps or detailed understanding of mosquito ecology, and it also results in immediate vector control through habitat mitigation. Local entomological surveillance programs can also use citizen science reports in the context of their rigorous surveillance to identify potential hotspots for investigation.

For future citizen science efforts directed toward detection or monitoring of *An. stephensi*, we recommend that at least six photos be taken per larva (two each of full body, head/thorax, and terminal abdominal segments), to ensure that all aspects of the anatomy are captured in-focus, as well as to generate a consensus of AI classifications (see third link below). For adult *Anopheles* found in urban or livestock settings, a smartphone equipped with a 60× clip-on lens can also provide sufficient magnification to capture the diagnostic spots on the wings and palps of *An. stephensi* (Figure 1 [17]). In the absence of a 60× clip-on lens, a high-powered magnifying glass or reversed binoculars can serve as alternatives for photographing larval or adult mosquito anatomy.

Such deployment of existing technologies to conduct enhanced monitoring of invasive and vector mosquitoes may continue to prove useful for targeted surveillance stratification, and as a local or regional launching point for national surveillance programs. Toward that end, we have also integrated multiple systems: our AI algorithms automatically analyze uploaded photos from the Mosquito Habitat Mapper, iNaturalist, and Mosquito Alert data streams to provide a real-time early warning system for larval and adult *An. stephensi*. To help further scale these surveillance efforts for others in Africa [17] and beyond, we provide the following free resources (all accessed on 7 May 2025):

1. mosquitodashboard.org: Global Mosquito Observations Dashboard (GMOD), a mapping interface for visualizing and downloading mosquito data from four citizen science platforms [18,23], including the GLOBE Observer app’s Mosquito Habitat Mapper and Land Cover tools;
2. mosquitoID.org: AI tools for identifying both larval and adult *An. stephensi* [55], as well as identifying the anatomy of any larval mosquitoes [20] and the gonotrophic stage of any adult mosquitoes [56].
3. observer.globe.gov: GLOBE Observer app’s Mosquito Habitat Mapper tutorials and other materials, including protocol eTraining modules [57,58];

We would also like to echo previous guidelines that any *Anopheles* spp. larvae found in artificial containers should be retained and/or reared for identification, and any unexpected increase in malaria cases—especially in an urban setting—should induce larval surveillance for *An. stephensi* and consideration of this species as a potential cause [14,16]. Additionally, more research is required to elucidate the taxonomic distribution of dark testes among *Anopheles* spp.

We also encourage the collection of adult samples of suspect *An. stephensi* in new areas when possible. When genetic analysis is not feasible and manual morphological identification is required, we recommend keys for *Anopheles* in the Afrotropical Region [59] and Madagascar [60], as well as reference images of diagnostic features for *An. stephensi* specifically (Figure 1 [17], Figures 3 and 4 [61]).

## 5. Conclusions

Given that the tire larva was disposed of years prior, we can never know the exact species with absolute, molecular certainty. However, all available evidence suggests that the mosquito may be *An. stephensi*, as it is an anopheline larva (1) from an artificial container in an urban setting; (2) found with and near >131 other anopheline larvae across multiple other types of artificial containers (meaning that the observation was not just a single isolated incident); (3) in a region both suitable and high risk for *An. stephensi* invasion; (4) with dark spots in segment VI identical to the testes of *An. stephensi* (and highlighted by the XAI heat maps); and critically, (5) all 11 of our AI models predicted the species to be *An. stephensi* with very high confidence and accuracy, and included a very low false positive rate (<1%); and (6) no other species of anopheline larvae were detected in any of the ~2 K artificial containers sampled during the subsequent year-long surveillance in the surrounding area and all six districts of Antananarivo.

Together, this case study demonstrates the promise of artificial intelligence for detecting artificial container-breeding disease vectors, especially when integrated with global citizen science efforts implemented via top-down and bottom-up approaches. Ultimately, our aim is that these next-generation digital tools and resources will be useful to communities and control programs for combating the spread of *An. stephensi* and malaria across the globe.

## Figures and Tables

**Table 1:** Sex model specimens (pre-augmentation).

Sex	Training		Validation		Testing	
	Images	Specimens	Images	Specimens	Images	Specimens
female	101	11	23	6	36	6
male	184	18	40	12	74	11

**Table 2:** Species model results, ranked by classification confidence of the tire larva.

Architecture	Model	Classes	Accuracy (Val) %	Accuracy (Test) %	Conf %	Classification
EfficientNet-B0	6-class	(*ara, gam, ste*) × (L3, L4)	96.30	95.83	99.34	*An. stephensi*
EfficientNet-B0	2-class, L4	*gam*, *ste*	100	91.67	99.27	*An. stephensi*
EfficientNet-B0	3-class	*ara*, *gam*, *ste*	96.30	91.67	98.61	*An. stephensi*
EfficientNet-B4	8-class	(*ara, fun*, *gam, ste*) × (L3, L4)	97.22	97.92	97.90	*An. stephensi*
EfficientNet-B4	10-class	(*alb, ara, fun*, *gam, ste*) × (L3, L4)	97.78	98.33	96.29	*An. stephensi*
Inception-ResNet-V2	4-class	(*gam*, *ste* × (L3, L4)	97.22	97.92	95.19	*An. stephensi*
EfficientNet-B4	16-class	(*tar, aeg, qui, alb, ara, fun*, *gam, ste*) × (L3, L4)	97.92	92.96	90.64	*An. stephensi*
EfficientNet-B4	14-class	(*aeg, qui, alb, ara, fun*, *gam, ste*) × (L3, L4)	99.21	97.62	89.87	*An. stephensi*
EfficientNet-B4	12-class	(*qui, alb, ara, fun*, *gam, ste*) × (L3, L4)	99.03	95.14	88.86	*An. stephensi*
EfficientNet-B0	4-class	(*gam*, *ste*) × (L3, L4)	97.22	93.75	88.83	*An. stephensi*
EfficientNet-B0	2-class, L3	*gam*, *ste*	100	100	67.95	*An. stephensi*
EfficientNet-B0	genus	*Anopheles*, not *Anopheles*	100	100	99.09	*Anopheles*

Only taxonomic (i.e., not including instar) classifications were used to calculate the validation and testing accuracies for each model, as well as the prediction confidence (conf) for the tire larva specifically. The genus model used all eight species. *aeg = Ae. aegypti*, *alb* = *Ae. albopictus*, *ara* = *An. arabiensis*, *fun* = *An. funestus*, *gam* = *An. gambiae*, *ste* = *An. stephensi*, *qui = Cx. quinquefasciatus*, *tar = Cx. tarsalis*, L3 = third instar, L4 = fourth instar.

**Table 3:** EfficientNet-B0 6-class species model results for the tire larva, ranked by confidence.

Class	Confidence (%)	
	Species and Instar	Species-Only
*An. stephensi* L4	82.56%	99.34%
*An. stephensi* L3	16.78%	
*An. arabiensis* L4	5.59 × 10^−1^	6.42 × 10^−1^
*An. arabiensis* L3	8.34 × 10^−2^	
*An. gambiae* L4	9.42 × 10^−3^	9.65 × 10^−3^
*An. gambiae* L3	2.24 × 10^−4^	

**Table 4:** Sex model results.

Set	Accuracy	F1-Score	Precision	Sensitivity	Specificity	MACC ^1^
Validation	95.44	96.49	94.33	98.75	89.67	90.18
Test	84.89	89.20	85.92	92.74	68.75	64.70

^1^ mean average correlation coefficient.

## Data Availability

GLOBE Observer data are publicly available at https://www.globe.gov/globe-data (accessed on 7 May 2025). Raw data analyzed in this project were accessed from this location. The Python code to read, analyze, and visualize GLOBE data for this article, as well as the analyzed data sets, are available on https://github.com/IGES-Geospatial (accessed on 7 May 2025). Dashboard access to Mosquito Habitat Mapper and Land Cover data is available at https://geospatial.strategies.org/ (accessed on 7 May 2025), as well as on the GMOD available at http://www.mosquitodashboard.org (accessed on 7 May 2025). To best enable re-use with attribution, all data products from the Earth System Exploration Portal are licensed with a Creative Commons Attribution 2.0 Generic (CC BY 2.0) data license.

## Abbreviations

The following abbreviations are used in this manuscript:

AI	artificial intelligence
CAM	class activation map
CDC CNN	Centers for Disease Control and Prevention Convolutional neural network
GLOBE	Global Learning and Observations to Benefit the Environment
GMOD	Global Mosquito Observations Dashboard
Grad-CAM	gradient-weighted class activation map
L1–L4	larval instar stages, one to four
MACC	mean average correlation coefficient
NASA	National Aeronautics and Space Administration
UA	University of Antananarivo
USF	University of South Florida
XAI	explainable artificial intelligence
WHO	World Health Organization

## Appendix A

**Table A1:** Species model architecture.

Layer	Input Size	Output Size
Base CNN blocks	224, 224, 3	7, 7, 1280
GlobalAveragePooling2D	7, 7, 1280	1280
dense 1 (Dense)	1280	256
batchNorm1 (BatchNormalization)	256	256
dropout 1 (Dropout)	256	256
dense 2 (Dense)	256	128
batchNorm2 (BatchNormalization)	128	128
dropout 2 (Dropout)	128	128
dense 3 (Dense)	128	64
batchNorm3 (BatchNormalization)	64	64
dropout 3 (Dropout)	64	64
concatenate_1 (Concatenate)	256, 128, 64	448
dense 4 (Dense)	448	6

**Table A2:** Species model hyperparameters.

Hyperparameter	Value
Loss	Sparse Categorical Cross-entropy
Optimizer	Adam Optimizer
Batch Size	16
Epochs	600
Learning-rate	1 × 10^−5^

**Table A3:** Sex model architecture.

Layer	Input Size	Output Size
Xception Conv blocks	299, 299, 3	7, 7, 512
global average pooling2d	7, 7, 512	512
dense 1 (Dense)	512	256
dropout 1 (Dropout)	256	256
dense 2 (Dense)	256	128
dropout 2 (Dropout)	128	128
dense 3 (Dense)	128	64
dropout 3 (Dropout)	64	64
dense 4 (Dense)	64	2

**Table A4:** Sex model hyperparameters.

Hyperparameter	Value
Loss	Binary Cross entropy
Optimizer	Adam Optimizer
Momentum	0.9
Epochs	1100
Learning-rate	0.0001
